# Neuropsychiatric Manifestation of Addison's Disease: A Rare Case Report

**DOI:** 10.7759/cureus.4356

**Published:** 2019-04-01

**Authors:** Maham Munawar, Pulwasha M Iftikhar, Choudhary A Hasan, Chaudhry Saad Sohail, Syed W Rizvi

**Affiliations:** 1 Internal Medicine, Dow University of Health Sciences (DUHS), Karachi, PAK; 2 Public Health, St. John's University, New York, USA; 3 Internal Medicine, Banner University Medical Center, Tucson, USA; 4 Internal Medicine, University of Medicine and Dentistry of New Jersey, Newark, USA

**Keywords:** psychosis, neuropsychiatric symptoms, lack of motivation, sleep disturbance, hyperpigmentation, hyponatremia, cortisol, addison’s disease, adrenal insufficiency, disorientation

## Abstract

Addison’s disease (AD), also known as primary adrenal insufficiency, is a rare autoimmune disorder affecting males and females equally. The most common cause of AD is autoimmune adrenalitis and other causes include metastatic cancers, tuberculosis and acquired immunodeficiency syndrome. AD presents with a wide variety of signs and symptoms and thus, making a diagnosis challenging. The common symptoms of this disease include weakness and fatigability, orthostatic hypotension, nausea, vomiting, diarrhea, anorexia and weight loss. Addison’s disease often presented with other autoimmune disorders, such as autoimmune polyglandular syndrome. We herein report a case of a patient who presented in a hospital emergency department, with Addisonian crisis and predominant neuropsychiatric manifestation. On review of the patient's history, combined with biochemical testing, a diagnosis of Addison’s disease was established. This type of presentation is relatively uncommon.

## Introduction

Addison’s disease (AD), also known as primary adrenal insufficiency, is a rare autoimmune disorder affecting males and females equally, with a prevalence rate of 100-140 cases per million [1-2]. AD is characterized by damage to the adrenal glands leading to insufficient cortisol, aldosterone and androgen production. Addisonian crisis is often the first clinical presentation in the majority of cases, due to the delay in diagnosis and treatment [3]. Chronic fatigue, vomiting, weight loss, anorexia, hypotension, hyponatremia, hyperkalemia, hypoglycemia and generalized intraoral and extraoral skin pigmentation (bronzing of skin), are the common signs and symptoms of AD. Neuropsychiatric symptoms include psychosis, mood disturbances. Motivation and behavior during the crisis are two of the unusual presentations of AD, hence making the diagnosis more challenging [4-6]. In this case report we present a case of AD with neuropsychiatric manifestations and Addisonian crises.

## Case presentation

A 32-year-old female presented to the emergency department with a four-month history of confusion, psychosis, slurred speech, nausea, vomiting and dizziness. Her symptoms included abdominal pain, headache, and depressed mood, lack of motivation and concentration, anorexia and associated 10 pounds weight loss in the prior three months. She had lost interest in her daily activities, and suffered considerable personality changes. All her symptoms were aggravated during periods of emotional and physical stress at her workplace, to the extent that she experienced episodes of panic attacks and insomnia. She consulted a psychologist for her depression and psychological symptoms, and was advised to engage in breathing exercises and yoga, neither of which improved her symptoms. On clinical examination, her pulse was 110 beats/min and blood pressure (BP) was 90/60 supine, thin brittle nails, scanty body hair, hyperpigmented knuckles, elbows and intraoral pigmentation of buccal mucosa. Laboratory investigations revealed anemia with hemoglobin level of 7.6 mg/dl, normal red blood cell morphology, erythrocyte sedimentation rate (ESR) 60 mm/h, and fasting blood sugar of 80 mg/dl. Her metabolic profile, including serum urea, creatinine, and electrolytes were within normal range. Mantoux tuberculin skin test was negative and her chest radiograph ruled out tuberculosis. Her thyroid and parathyroid hormone profiles were normal. Her morning serum cortisol (4.54 micrograms/dl, N: 4.3-22.4 micrograms/dl) and serum aldosterone levels (27.50 pg/dl, N: 25-315 pg/dl) were also normal. An ACTH stimulation test showed poor response (prestimulation level of 14.64 micrograms/dl, poststimulation level of 13.87 micrograms/dl and normal expected rise of 10 micrograms/dl). Antinuclear antibodies, rheumatoid factor, hepatitis B, hepatitis C and HIV were negative. Based on the patient’s self-reported history, clinical history and laboratory investigations, the diagnosis of Addison’s disease was established. She was managed with IV hydrocortisone, parenteral fluids, and glucose supplements during her Addisonian crisis. She recovered after two days of hospitalization. After a complete evaluation, cortisol replacement in the form of methylprednisolone 10 mg in the morning and 5 mg in evening was started. Her skin pigmentation, appetite, neuropsychiatric symptoms, mood and sleep disturbance have improved with cortisol replacement therapy, and she feels more motivated at her workplace.

## Discussion

Adrenal insufficiency is predominantly classified as primary adrenal insufficiency, in which the adrenal gland is dysfunctional primarily or secondarily, whereas central adrenal insufficiency is due to lack of secretion of corticotropin releasing hormone from the hypothalamus or adrenocorticotropic releasing hormone from the pituitary gland [6,7]. Addison’s disease was first described by Thomas Addison in 1855 and he also mentioned that AD patients might present with “attacks of giddiness, anxiety in the face, and delirium” [7-9]. Addison’s disease has unusual presentation with diverse signs and symptoms, hence confusing and often delaying definite diagnosis. However, the usual presenting symptoms include weakness, weight loss (>90%), gastrointestinal complaints (>80%), body aches (18%), salt craving, hyperpigmentation, syncope and disorientation (12-15%) [9,10]. Psychiatric symptoms include sleep disturbances, mood and behavior changes with decreased motivation. The exact etiology of the neuropsychiatric symptoms corresponding with AD are not specified, yet could be related to the disruption of electrophysiological, metabolic activities and electrolyte imbalance (hyponatremia) [10]. This case presented with the history of altered sleep pattern, mood and behavioral changes, which was first misdiagnosed as depression. Her vague symptoms and the nature of her work, resulted in a delayed diagnosis. A study by Cleghorn and Pattee showed that AD can present with mood, behavior changes, acute organic brain syndrome, psychosis and cognitive decline, which are often mistreated and it can lead to Addisonian crisis [11]. Moreover, since 1940, 25 cases of Addison’s disease with predominant psychiatric symptoms have been reported [7, 9-11]. Study conducted by Iwata et al. showed that in some AD cases, patient presents with the neuropsychiatric symptoms as the sole presentation, even though such symptoms are not common in the initial phase of the disease [12]. The hallmark sign of Addison’s disease is hyperpigmentation of the skin due to excessive ACTH production, which in our case served as an important diagnostic sign. Initially, the patient presented with classical symptoms of mood disorder, which is exceedingly common in developed countries, but due to delay in exact diagnosis and appropriate treatment, her symptoms were aggravated, and she presented in the emergency department with Addisonian crisis. Hyponatremia, hypoglycemia, and hyperpigmentation were all important markers for the diagnosis of Addison’s disease. Her condition showed remarkable improvement on the administration of IV hydrocortisone. This case emphasizes the psychiatric manifestations associated with Addison’s disease, which are often ignored. These manifestations although rare, and in some cases be the only primary symptoms in AD. Hence any patient, presenting with indistinct signs and symptoms, should be promptly tested for autoimmune disorders in order to prevent the Addisonian emergency.

## Conclusions

Adrenal insufficiency is an imperative disorder that needs early medical attention and appropriate treatment in order to prevent complication of this disorder. Physicians must be incredibly knowledgeable with the neuropsychiatric symptoms of AD. These symptoms are sometimes the first manifestation and is often ignored that causes rapid progression. The disease has a good prognosis with quick improvement in neuropsychiatric symptoms if diagnosed early.
